# Modernizing biomolecular NMR: The POKY suite

**DOI:** 10.1016/j.jbc.2026.111246

**Published:** 2026-02-05

**Authors:** Abigail Chiu, Woonghee Lee

**Affiliations:** 1 Department of Integrative Biology, University of Colorado Denver, Colorado, USA; 2 Department of Chemistry, University of Colorado Denver, Colorado, USA; 3 Center for Advanced Computational Molecular Science, University of Colorado Denver, Colorado, USA

**Keywords:** structural biology, NMR spectroscopy, resonance assignments, structure determination, biomolecular NMR, metabolomics, software, POKY

## Abstract

Biomolecular NMR spectroscopy has been a keystone in structural biology for decades. It can provide unique, atomic-level insights into protein dynamics, interactions, and conformational ensembles. However, its complex workflows and fragmented data analysis pipelines are often perceived as significant barriers to entry. This review highlights the POKY suite as a comprehensive solution that modernizes and streamlines the entire biomolecular NMR process. From spectral processing to structure calculation, POKY creates a single user-friendly cyber infrastructure for a seamless and efficient NMR data analysis environment. A key aspect of its design is the integration of various artificial intelligence components to streamline complex tasks and reduce user burden, such as automation, unsupervised learning, and more. While recent advances within *in silico* artificial intelligence prediction models have raised questions about the role of experimental data, POKY provides a clear answer. This ecosystem can create a powerful synergy between the experimental data with structure prediction. Modernizing the experimental workflow, POKY makes NMR more accessible and powerful, reinforcing its vital role in structural biology.

From energy storage to genetic inheritance, biomolecules, such as proteins, carbohydrates, and lipids, are essential in sustaining biological systems. Their 3D structures and dynamic behaviors can determine their function, which is the central aim of structural biology. This idea of studying structure to understand function was introduced after a defining event in the 1990s. This was the Human Genome Project (HGP) that started in 1990 by the National Institutes of Health (NIH) (1). Over 13 years, the HGP successfully mapped and sequenced the entire human genome (approximately 3 billion pairs), marking the beginning of the genomics era. Yet, despite this monumental achievement, the biological roles of many genes remained unclear because sequencing only provides the genetic blueprint and not functional or structural context. To address this gap, the concept of “structural genomics” was introduced in 1996 to focus on determining the function of the proteins that the genes encoded (2). This initiative flourished until 2015 and led to the discovery of around 14,000 new protein structures encoded by the HGP-discovered genes (3, 4). From the 3D arrangement of protein structures, functional information, such as active sites and molecular interactions, could be learned. By 2023, around 200,000 experimentally determined protein structures have been determined (5). Structural biology has since been pivotal not only in understanding proteins and nucleic acids but also in illustrating how metabolites interact with these macromolecules to shape cellular metabolism (6). Specifically, solving the structures of metabolic enzymes bound to their substrates or inhibitors can map atomic interactions in the metabolic pathways. By studying how specific molecular interactions drive biological processes, structural biology has shown that living systems function as dynamic molecular networks rather than static collections of parts.

To investigate how individual proteins function in these dynamic networks, knowing their 3D structure is the essential first step. Advances in structural biology have led to several powerful techniques for protein structure determination with distinct advantages and limitations. Among the most widely used are X-ray crystallography, cryo-EM, and NMR spectroscopy. X-ray crystallography, a fundamental technique in the field, can provide atomic-scale resolution for well-ordered proteins. However, one major drawback is the requirement for sample crystallization. This process is not only challenging for large or flexible proteins since they are less likely to form ordered and uniform lattice structures (7, 8) but also can alter the protein’s native conformation because the dense crystal packing can induce nonphysiological arrangements (9). In addition, the high-intensity X-ray can cause radiation damage to the sample because the X-ray ionizes atoms in the crystal, which creates reactive molecules that can break the chemical bonds in the protein sample (10). Consequently, the resulting electron density map is a static snapshot of the protein's average conformation (11). This map with the nonphysiological constraints from crystal packing can limit the protein’s natural flexibility and make it more difficult to study its conformational dynamics (11). On the other hand, cryo-EM is a powerful alternative that does not require crystallization, making it especially useful for membrane proteins and large protein complexes. A key advantage of this is its ability to show multiple conformational states from a sample. Still, cryo-EM struggles with proteins smaller than 50 kDa. These smaller targets may be more challenging to model because they have fewer atoms to generate strong signals, leading to a poor signal-to-noise ratio. This low contrast could challenge reconstruction software to accurately align individual particle images (12, 13). Moreover, its sample preparation can introduce nonphysiological structural heterogeneity (14), and flexible regions often resist alignment and blur the final 3D model (15).

Last, NMR spectroscopy is a versatile technique that can not only determine structures but also explore protein dynamics and interactions under near cellular–physiological states. This method determines the interactions of atomic nuclei with their surroundings by probing nuclear spins. To achieve this, samples are subjected to a strong magnetic field, which aligns the nuclear spins to create a bulk polarization. Radiofrequency pulses measure this polarization to enable the study of specific interactions *via* dedicated correlation maps (*i.e*., spectra). These resulting spectra can reveal the number and types of atoms, connectivity, and spatial arrangement. Still, determining a 3D structure requires a multistep process. The initial step is resonance assignment, where through-bond correlation experiments (such as ^1^H–^15^N heteronuclear single quantum coherence [HSQC] and 3D CBCA(CO)NH) are used to identify each signal to an atom in the protein sequence (16, 17). Once those resonances are assigned, through-space experiments like NOESY can identify protons that are physically close to each other in the final folded structure (16, 17). These experiments provide the geometric “restraints” used to calculate the 3D model. The most critical information is derived from NOE, which provides through-space distance restraints by measuring dipolar interactions; scalar couplings (J-couplings), which help define dihedral angle restraints (*i.e*., the local bond angle) from chemical shifts; and residual dipolar coupling, which provides long-range orientation information to show how the parts of a protein are aligned to one another. These experimentally derived parameters are then incorporated as potential energy terms during structural calculation methods, such as simulated annealing (16, 18).

Beyond structure determination, NMR spectroscopy can provide protein dynamic information across a vast range of timescales, from picoseconds to seconds (19). For fast motions (picosecond to nanosecond), NMR relaxation measurements (R_1_, R_2_, and NOE) quantify the rapid local flexibility in the backbone and side chains of a protein (19). For slower motions (microsecond to millisecond), relaxation dispersion experiments can probe slower or more functionally significant movements, such as enzyme activity (19). For global changes (seconds to hours), hydrogen–deuterium exchange can capture large-scale conformational changes over long periods (19). For protein–ligand interactions, chemical shift perturbation (CSP) methods can detect shifts in resonance signals upon ligand binding to a protein because this binding causes alterations in the magnetic environment of the amino acids at the binding interface (20). This CSP analysis is typically performed as an NMR titration experiment. In this experiment, the ligand is gradually added to the protein sample, and the shift progression of the involved signals is monitored. By tracking the magnitude of these peak shifts as a function of ligand concentration, researchers can precisely map the residues that form the binding site and calculate the binding affinity (*K*_*d*_) (20).

This suite of experiments makes solution-NMR methods well suited for small- to medium-sized soluble proteins and provides detailed information about structure, binding interactions with partners (*e.g*., proteins, nucleic acids, or metal ions), conformational changes, flexible regions, and druggable sites (21). Still, solution NMR is generally limited to soluble proteins smaller than approximately 25 kDa because the technique relies on the rapid rotational diffusion of the proteins. So, larger proteins tend to diffuse more slowly, which can cause faster signal decay that leads to larger and flatter signals (22). Furthermore, because these proteins have thousands of atoms, the signal for each of these atoms can easily overlap in a spectral window, causing complicated signal assignment (22). To overcome the size barrier, advanced solution-NMR methods, such as transverse relaxation-optimized spectroscopy, are employed to study large proteins by reducing relaxation-induced signal loss (23). Solid-state NMR (ssNMR) utilizing magic angle spinning is used for larger, insoluble, or heterogeneous systems, such as nanocrystalline/microcrystalline globular proteins and fibrils, because it does not rely on the rapid isotropic rotational diffusion of the sample (24). In addition, to further enhance ssNMR capabilities, a new pulse program called the Adiabatic Linearly Frequency Swept recoupling scheme was recently developed to perform measurement of long-range distances between atomic nuclei that are normally hidden by signals because of atomic nuclei with short distances (25). Still, given the complexity of protein structure and function, no single technique is universally optimal. Instead, a multimodal approach among X-ray, cryo-EM, NMR, and even artificial intelligence (AI) may offer the most comprehensive structural information to build more accurate macromolecule models.

Complementing these experimental techniques, AI has recently advanced the field with sophisticated protein structure prediction techniques. While the term “AI” is often synonymous with complex deep learning, the term historically encompassed any computational method designed to perform tasks that mimic human reasoning and problem solving (26, 27). The term “AI” is a broad definition that includes foundational automation, search algorithms, and comparative analyses that paved the way for modern approaches (https://ibm.com/think/topics/artificial-intelligence). For instance, early computational methods focused on comparative modeling and *ab initio* prediction (28). Comparative modeling identifies homologous proteins with known structures by aligning the target protein sequence with the template sequence (29). *Ab initio* prediction uses only the protein sequence to search for the lowest free-energy structure from its conformational space (30). Tools, such as MODELLER (29), Rosetta (31), and I-TASSER (32), pioneered the field by predicting 3D structures directly from their amino acid sequences. By integrating NMR-derived data, hybrid tools like CS-ROSETTA (33) and PACSY-based POND-PRED (34) improved predictive accuracy with the incorporation of experimental constraints. To promote the importance of benchmarking, which is the process of evaluating prediction tools, Critical Assessment of Structure Prediction competition held biennially has been accelerating progress in this area (35). The real evolution came with the integration of deep learning. Early rule-based AI systems, like IBM’s Deep Blue, a chess-playing supercomputer that used computational search and symbolic reasoning without machine learning capabilities, laid the foundation for more powerful models (36). DeepMind’s AlphaGo, an AI program that played the complex traditional board game, Go, used deep neural networks and reinforcement learning on a massive training dataset. This marked a significant milestone in AI (37). Building on this, DeepMind released AlphaFold 1 and 2, with AlphaFold 2 achieving unprecedented accuracy at the CASP14 with a 92.4 Global Distance Test in protein structure prediction by employing deep learning, evolutionary data, and attention-based neural networks (38). The user-friendly interface provided by ColabFold, a free cloud-based AlphaFold using Google Colab’s notebook environment, played a crucial role in AlphaFold’s widespread adoption by offering a simplified interface and workflow that no longer required users to download numerous software packages and dependencies (39). Most recently, AlphaFold 3 has extended its capabilities beyond proteins to include nucleic acids and ligands for complex biomolecular modeling (40).

In recognition of this transformative work, the 2024 Nobel Prize in Chemistry was awarded to contributions on computational protein design and protein structure prediction (https://nature.com/collections/edjcfdihdi). Despite this success, AI-based modeling still faces notable limitations. Model development remains computationally intensive and time consuming. In many cases, structural databases are incomplete, limited in size, or biased, which can restrict model training quality and compromise prediction accuracy. Moreover, proteins are dynamic molecules that can have conformational changes across different functional states. While AI prediction models typically provide only a single static snapshot, this is an active area of research. New methods, such as BioEmu (41), AlphaFold-Cluster (42), and AlphaFold2-RAVE (43) are attempting to generate more dynamic protein models. This trend highlights a unique and critical role for NMR spectroscopy in validation (44). There have been studies emerging to not only validate and refine predicted models (45, 46) but also offer the potential of hybrid methods that combine the AI predictions with experimental methods like NMR spectroscopy (47, 48, 49, 50).

While NMR spectroscopy can provide important, atomic-level insights about biomolecular structures, dynamics, and interactions, its complex analysis often creates a steep learning curve that limits its accessibility to a wider scientific community. Challenges may arise at every stage of its process, from data processing and peak picking to resonance assignment and structure calculation. Manually performing these procedures can be long, tedious, and prone to errors. Furthermore, spectra often suffer from severe crowding. So, assigning thousands of individual resonance signals to specific atoms in a protein sequence is a nontrivial puzzle that often gets complicated by signal overlap. While early computational algorithms offered some help, they still require significant parameter tuning by a human expert. This bottleneck led to the adoption of AI, specifically machine learning, as early as 1971, where a pattern recognition method was applied to identify molecular structure features from NMR spectra (51).

Since that early work, significant developments have occurred not only in NMR instrumentation with magnets now beyond 1 GHz for improved sensitivity and resolution but also in AI-driven NMR data analysis for a more efficient and reliable analytical process (52). Still, the software development for structure calculation and extracting scientific meanings is relatively slow compared with the development of instruments and pulse programs. A primary issue is that the typical NMR workflow is neither straightforward nor integrated. Not only is the learning curve for each step steep on its own, but researchers must also master a collection of disparate programs, where each has its own interface and user philosophy. To illustrate, after collecting data, users typically need to process their raw time-domain data to a frequency-domain spectrum using programs, such as NMRPipe (53) or Bruker Topspin. Then, the processed spectrum is exported to a different program, such as Collaborative Computing Project for NMR analysis (54) or NMRFx (55), for visualization and resonance assignment. Finally, the assignment lists and peak tables are fed into yet another software, such as XPLOR-NIH (56), for 3D structure calculation. These existing programs may require users to follow their specific file format and workflow or have preferences on developing tools for other types of molecules, such as nucleic acids. They can make the learning curve higher because users need to perform additional steps to adapt their workflow to a software, which can make their results more error prone. This disconnected process is notoriously inefficient and may take up to years to complete. Users would have to learn multiple complex interfaces, constantly manage file format conversions, and manually transfer data. Because this process can create numerous opportunities for errors, efficient structure determination often demands extensive expertise and a large number of computational resources. This lack of an accessible, integrated, and user-friendly software platform has created a steep learning curve that deters many researchers from fully embracing NMR-based techniques. This is where the POKY suite can come in. The POKY suite focuses on providing a single user-friendly platform that integrates the entire NMR analysis workflow. Instead of replacing all existing tools, POKY integrates well-known programs, such as NMRPipe (53) and XPLOR-NIH (56), into one environment. POKY streamlines the process not only through a simple interface for file conversions through easy data import and export but also gives researchers the freedom to mix-and-match tools for their analysis. For beginners, POKY provides a recommended workflow with supported documentation, tutorial videos, and an active user group that shares troubleshooting strategies and tips (NMR POKY/SPARKY USER GROUP; https://groups.google.com/g/nmr-sparky). Moreover, POKY is built to handle computationally intensive jobs through high-performance servers, such as graphical processing units. As POKY is constantly improving, it is well suited not only for structural biology but also for other rapidly evolving fields, such as metabolomics and ssNMR.

## Evolution of POKY

**Figure 1:**
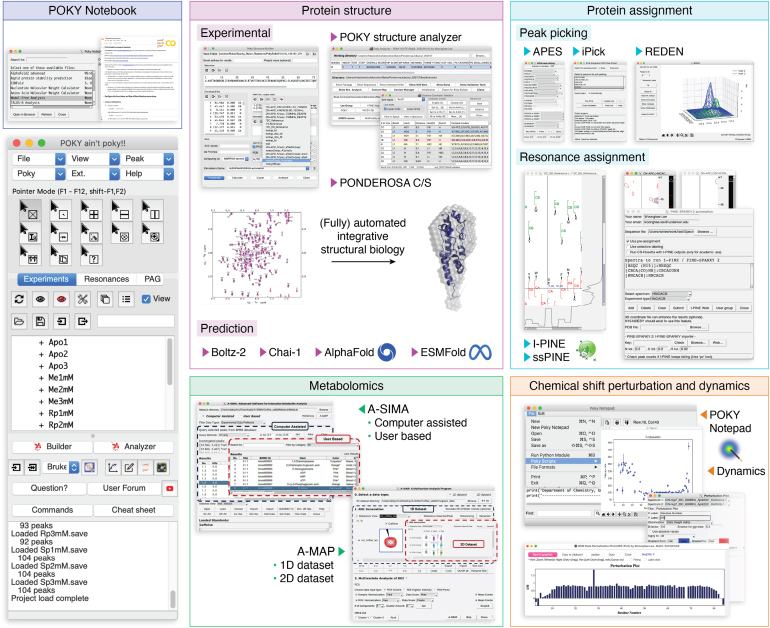
**The POKY suite contains various tools for many different NMR analysis.** The image on the *bottom left* is the POKY suite graphical user interface (GUI). From the POKY GUI, users can access programs for analysis, such as protein assignment, protein structure calculation, metabolite analysis, chemical shift perturbation, and dynamics studies. Many general tools are provided in the form of GUI. More specific analytical tools are provided in a separate GUI called POKY Notebook that contains links to their respective Google Colab notebooks.

The development of the POKY software suite can be traced back to the UCSF-SPARKY in the 1990s from the Kuntz and Ferrin groups, followed by major enhancements under the NMRFAM-SPARKY in the 2010s (57), and recently released the POKY suite in 2021 (58). Designed as a comprehensive ecosystem, POKY incorporates a range of powerful tools, including the original PONDEROSA automated structure calculation program (59) and its successor, PONDEROSA-C/S (60). The PONDEROSA-C/S rebuilt the original PONDEROSA workflow into a more powerful client–server system for remote computation to support end-to-end structural analysis with a unified interface (Fig. 1) (61). The suite has already proven effective across diverse applications, such as automated pick peaking and assignments in a multidomain protein study, a structure determination study of high-energy states in a dynamic protein (62), and analysis of lignins (63). By building an integrated AI-assisted cyber infrastructure, POKY provides various levels of interfaces to process complex NMR data with greater efficiency and flexibility.

**Figure 2:**
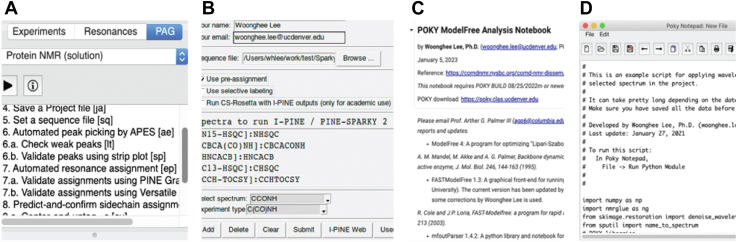
**Four different examples of the types of user interface in POKY.***A*, POKY Automation Guide (PAG) for typical solution protein NMR. *B*, popular PINE-SPARKY.2 graphical user interface (GUI) extension for automatic assignments. *C*, POKY ModelFree Analysis Notebook in Google Collaboratory for protein dynamics. *D*, a wavelet denoising script in POKY Notepad. PINE, Probabilistic Interaction Network of Evidence.

POKY provides four tiers of user interfaces to accommodate users of varying expertise. The first, POKY Automation Guide, offers object-oriented user interfaces providing step-by-step guided automations (Fig. 2*A*). POKY Extensions simplify tasks with high-level push button functionality (Fig. 2*B*), whereas POKY Notebooks provide a Jupyter-based environment, an interactive platform of live code, texts, and visualizations, for intermediate users seeking customizable workflows (Fig. 2*C*). For advanced users, POKY Notepad offers a scripting interface with direct access to tools and updates *via* the platform’s GitHub repository (Fig. 2*D*). These diverse user interface options allow users to rapidly perform traditionally time-consuming tasks, such as peak picking, resonance assignment, and structure calculation. Each task can be accessed through drop-down menus or by typing two-letter codes in the POKY command window. With these user interface tiers, POKY becomes a user-friendly solution that connects high-performance instrumentation with accessible tools for a streamlined analysis. By lowering the technical and computational barriers to structural analysis, POKY fosters greater innovation and wider engagement across the biological sciences.

## Resonance assignments

**Figure 3:**
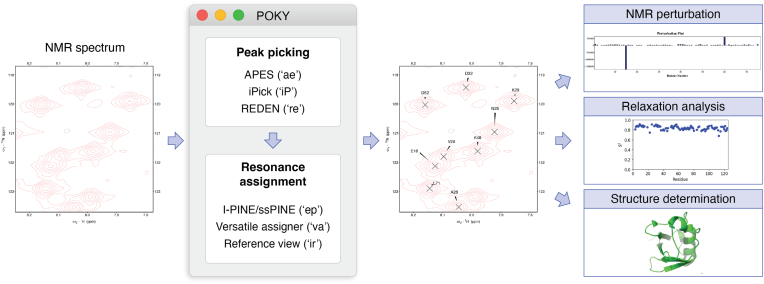
**Overall assignment workflow.** After processing the NMR spectrum, users can use POKY for peak picking *via* the tools, such as APES, iPick, and REDEN. For resonance assignment, users can perform automatic assignments with I-PINE/ssPINE (for solid-state NMR data), semiautomated assignments with versatile assigner, and manual assignments with a reference view. After assignments, users can perform NMR perturbation, relaxation analysis, or structure determination. All programs can be opened through the POKY main menu or type the two-letter code shortcut in POKY command window. I-PINE, Integrative Probabilistic Interaction Network of Evidence; ssPINE, solid-state Probabilistic Interaction Network of Evidence.

Accurate resonance assignment is crucial for understanding biomolecules by NMR spectroscopy, and it begins with obtaining the highest quality spectra possible. After data collection, the raw time-domain data must undergo a series of preprocessing procedures to prepare it for analysis. This process begins with refining the NMR time-domain data using methods such as apodization to enhance line shapes and improve signal-to-noise ratio (64), zero-filling to increase the number of spectral data points for smoother peaks (65), and linear prediction to correct truncated signals and improve spectral resolution (66, 67). After Fourier transformation, phasing and baseline correction are applied to produce a clean and flat baseline with correct peak shapes for more accurate peak assignment and integration (68). An example of this can be found in this study on disorder predictors for disordered proteins (69). They processed their data with apodization and zero filling using NMRPipe (53) and performed their analysis using NMRView (70). The methods used in this study can all be found in the POKY suite through the Data Processing option in the POKY Automation Guide. Aside from these processing tools, variation in chemical shifts can occur because of differences in factors, such as pH or temperature, between experiments (68). These variations can hinder the rest of the data analysis. As such, POKY offers a spectral alignment tool (accessed in POKY *via* two-letter code “*al*”) that can correct those minor variations. Once the spectral data are processed, the workflow continues to peak picking, resonance assignments, structure calculation, scripting, batching, and more. These procedure steps are commonly performed by various programs, as shown by this NMR structure determination study on a protein part of the Pfam protein family PF06042 (71). In this study, they used two automated chemical shift assignment programs (UNIO-MATCH 2.0.1 (72) and UNIO-ATNOS/ASCAN 2.0.1 (73)) for NOESY data assignment and confirmed using a different manual program, CARA (74). If users want to perform automated assignment and validate manually like this example study, POKY has tools for peak picking and resonance assignment in solution or ssNMR, as shown in Figure 3.

For automated peak picking, users can choose between APES (“*ae*”) for standard solution NMR experiments or iPick (“*iP*”) for either solution or ssNMR with more flexibility in terms of experiments (75). In cases where peaks are too close or partially overlapping, REDEN (“*r*”) can provide line shape simulation to search for the possibility of shouldering peaks (76). To visualize and manage the picked peaks, users can employ a peak list (“*l”*’) for an overview and a strip plot (“*sp*”) to inspect spectral strips for peak pair verification. After peak picking, POKY provides different pathways for resonance assignments to accommodate users with varying levels of expertise and desired control. For a full *predict-and-confirm* experience, POKY offers two distinct options. If a reference assignment is already available, the transfer and simulate assignment tool ("*ta*”) can add Biological Magnetic Resonance Bank or SHIFTX2 assignments (77, 78). Otherwise, the Probabilistic Interaction Network of Evidence (PINE)-SPARKY.2 module (“*ep*”) allows probabilistic assignment based on the provided protein sequence and a combination of 2D, 3D, and/or 4D experimental spectra (79, 80). The input values are sent to the Integrative PINE (I-PINE) web server, which is the exclusive version associated with POKY’s web resources and is still being actively worked on for more features. I-PINE performs the probabilistic assignments by analyzing sequence data and peak lists of multiple NMR experiments (81, 82). This is distinct from the older version submitted by NMRFAM-SPARKY, which is no longer updated. The resulting assignments can be evaluated using strip plot (“*sp*”) or the PINE-SPARKY package that maps assignments onto the protein sequence and associated molecular structure for intuitive visualization and graphical probabilistic verification (79). If users seek a more hands-on or guided experience, the Versatile Assigner (“*va*”) and Reference Views (“*ir*”) can be used. These tools enable semiautomated assignment by going through peak pairs interactively *via* the strip plot (83). For complete manual control, POKY also supports manual assignment (“*at*”), which gives flexibility for expert users. In both semiautomated and manual workflows, a new tool, TINTO, can be valuable, as it uses the idea of image similarity measure for resolving ambiguities and ensuring assignment continuity in case peak-based matching is difficult because of various reasons (84). In addition, POKY offers chemical shift–based secondary structure prediction tools. I-PINE web servers automatically results in a bundled report of not only I-PINE assignment probabilities but also Protein Energetic Conformational Analysis (PECAN; implemented in POKY as pyPECAN) for probabilistic secondary structure prediction (85), Linear Analysis of Chemical Shifts (LACS; pyLACS in POKY) for automated detection of assignment outliers and correction of errors in referencing results (86), TALOS-N for prediction of protein backbone and side-chain torsion angles from NMR chemical shifts (87), chemical shift index to identify protein secondary structures (88), GetSBY for secondary structure prediction using PACSY database (17), and PSI-PRED for protein secondary structure prediction based on position-specific scoring matrices (89). These analyses can also be performed independently for more direct control outside the automated I-PINE workflow. From initial peak picking to final assignment validation, POKY provides a complete solution for one of the most challenging tasks in NMR spectroscopy. From there, researchers can go into structure calculation, relaxation analysis, and NMR perturbation analysis.

## Structure prediction and determination

A fundamental goal to understanding the function of a protein in structural biology is to determine its structure. Like the other procedural steps, this step not only requires many independent software but also a significant number of computational resources. To demonstrate, a structure determination study of the RNA recognition domain in the METTL3 protein used XPLOR-NIH (56) with NOE distance restraints, backbone dihedral angle restraints from TALOS+ (90), and more to calculate and refine its structure (91). Two hundred fifty-six computational simulations were completed to create an ensemble of the lowest energy structures with the least number of violations. This process can take a very long time and may even fail if there is not enough computing power. Because structure calculation may require lots of computational power, POKY offers not only high-computing servers but also tools, including XPLOR-NIH (56) and TALOS-N (87), which has higher coverage, accuracy, and reliability than TALOS+ (92), for structure calculation and refinement. Furthermore, in specialized studies, such as this study on a helical membrane protein *via* polarization inversion with spin exchange at the magic angle (93), polar index slant angle (PISA)-POKY (“*PS*”), previously known as PISA-SPARKY (94), can perform exhaustive fitting using PISA-wheel models to find the precise tilt and rotation angles of α-helical segments of each peak in the 2D (^15^N–^1^H) polarization inversion with spin exchange at the magic angle separated local field spectrum. PISA-POKY can perform fitting and error analysis with the resulting orientational restraints and then be exported for full structure calculation.

**Figure 4:**
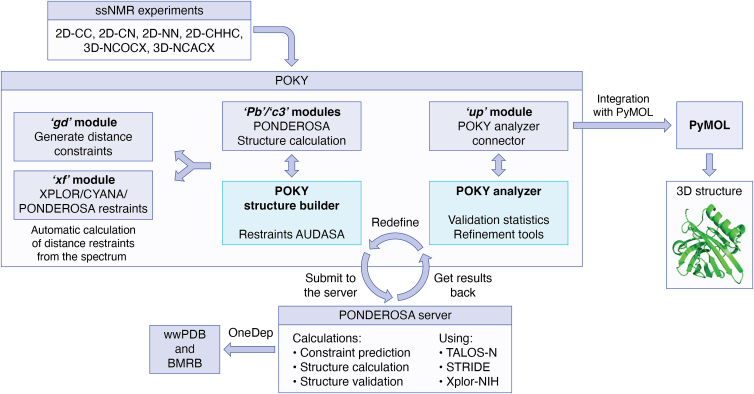
**A schematic diagram of the structure calculation tools in the POKY suite.** It shows the procedural steps to calculate 3D protein structure from ssNMR spectra. Once users have obtained their necessary NMR spectra and performed backbone and side-chain assignments, users have three possible pathways. One is a fully automated approach that uses POKY Structure Builder for automated 3D NOESY spectra assignment, distance restraint generation, and perform structure calculation. Second is a semiautomated approach to automatically generate distance restraints with assigned NOE peaks with the “*gd*” module and input the results into POKY structure builder to perform structure calculation. Third option is a more manual approach to generate restraints using the “*xf*” module, which will then also be used as input for POKY Structure Builder job submission. After submitting the distance restraints (if any), protein sequence, and chemical shift assignment to the PONDEROSA server, multiple servers will process the inputs, calculate the 3D structure, and estimate quality of structure using XPLOR-NIH, TALOS-N, and STRIDE for calculation. After getting results back, users can use POKY Analyzer or POKY Analyzer Connector (“*up*”) to validate and refine their structure. The structure can be viewed using PyMOL in POKY. This process is applicable to solution NMR data. ssNMR, solid-state NMR.

This PISA-POKY plug-in complements the general structure calculation workflow (Fig. 4) that begins with the POKY Structure Builder (95). Users can first prepare the essential inputs: protein sequence, chemical shift assignments of their backbone and side-chain spectra, and through-space NOESY data. Generating distance restraints from NOESY data can be done either automatically, semiautomatically, or manually. For manual or semiautomation, users can automatically generate distance restraints from assigned NOE peaks using the “Generate Distance Constraint” module (“*gd*”) or manually customize them using the “XPLOR/CYANA/PONDEROSA Restraints” plugin (“*xf*,” 60). The resulting file would then be uploaded into the POKY Structure Builder with protein sequence and chemical shift assignments to send a job to the PONDEROSA server for structure calculation. In full automation, users can just input their sequence and chemical shift assignments into the POKY Structure Builder with the “AUDANA/AUDASA automation” calculation option selected to automatically assign nonsequential crosspeaks in 3D-NOESY or long mixing-time ssNMR magic angle spinning spectra, generate distance constraints, and perform 3D structure calculation through iterative high-temperature molecular dynamics and simulated annealing (96). For distance restraints, the AUDANA (for solution NMR) or AUDASA (for ssNMR) algorithm not only searches the PACSY database (34) for homologous protein structures and their known 3D information but also uses ESMFold (97) to predict possible structure, if users choose the PACSY boost option and set the Protein Data Bank ID and chain to “AUTO” (95, 96). These predictions are then compared with experimental data to score and validate ambiguous NOE assignments. POKY has further enhanced POKY Structure Builder by allowing users to import their own predetermined or predicted structures in the Restraints section to help resolve ambiguities in NOE assignments. These validated restraints then guide the final structure refinement through iterative structure calculation. Alternatively, if there are sufficient restraints, users can select the “Constraints-only X” calculation option in the POKY Structure Builder to proceed directly to structure calculation. Once prepared, the data are submitted to the PONDEROSA server. Using XPLOR-NIH (56), TALOS-N (87), and STRIDE (98), the PONDEROSA server will perform iterative calculations and validations before returning the results *via* electronic mail. Finally, users can visualize the 3D structure, analyze validation statistics, and inspect restraint violations in the POKY Analyzer or the POKY Analyzer Connector (“*up*”). This provides a powerful iterative loop, allowing users to refine their constraints and resubmit jobs for further improvement. With the POKY Structure Builder and the POKY Analyzer/POKY Analyzer Connector (“*up*”), their combination can provide an efficient workflow for researchers to determine accurate 3D protein structures effortlessly. From data collection, local analysis, to structure calculation, POKY provides a robust and user-friendly platform to obtain high-quality protein structures from NMR data.

Aside from NMR-based structure determination, POKY also supports the latest advancements in protein structure prediction. POKY integrates cutting-edge protein prediction tools, such as AlphaFold2/3 (38), ESMFold (97), Boltz-2 (“*bz*,” 99), and Chai-1 (“*ch*,” 100). These tools apply deep learning models trained on large structural databases to predict 3D folds from just a protein sequence. Some programs, such as AF-Cluster (42) and ESMFold (97), are even expanding to subsampling and drug design. This inclusion in POKY allows for better accessibility *via* a user-friendly interface to these computationally demanding tools. This removes a significant burden on researchers to install the complex software and maintain the required graphics processing unit servers to run them. The resulting computed structure models can serve as starting models for refinement against experimental NMR data. Conversely, sparse or ambiguous NMR restraints can be used to crossvalidate the AI predictions. For example, users can load a predicted model into the POKY Structure Builder to back-calculate a theoretical NOESY spectrum that can then be directly compared with the experimental data. This can provide a robust validation since long-range distance restraints are critical for defining overall protein fold. By uniting prediction with experimental data and *in silico* prediction, researchers can tackle complex structural problems with a higher degree of confidence and efficiency.

## Protein dynamics and interactions

Proteins are not static but highly dynamic and rapidly fluctuate through a large amount of conformational substrates. Understanding these movements, known as protein dynamics, can provide crucial information about their function and behavior. A unique and captivating advantage of NMR is its ability to probe these dynamics experimentally. This can be seen in a study on the dynamic allostery in substrate binding by human thymidylate synthase (hTS) (101). Because there are not lots of existing software programs, researchers often must make their own scripts, such as the case in the hTs study, where they created their own in-house script for global analysis of Carr–Purcell–Meiboom–Gill and Chemical Exchange Saturation Transfer data. Fortunately, POKY offers tools for protein dynamics studies through four different graphical user interfaces (Fig. 2) and notably the POKY Notepad (“*Pn*”), which is a scripting hub for customizable analyses. Like the case study, the POKY Notepad has a ^15^N Carr–Purcell–Meiboom–Gill relaxation analysis script to study slower and functionally important motions in a protein. This technique can be used to study conformational exchange and structures of “invisible” states on the microsecond to millisecond timescale to give insights about structural flexibility and exchange processes like ligand binding and allosteric regulation (102, 103). For the hTS case study, this script could be used as a starting point for their three-state model, and then they could further the code in POKY Notepad for their analysis. For faster local dynamics studies, the POKY Notepad includes a wide range of scripts for standard relaxation analyses. Some of these scripts can automate the measurement of *R*_*1*_ (spin–lattice), *R*_*2*_ (spin–spin), and heteronuclear NOE rates to characterize fast protein motions on the picosecond-to-nanosecond timescale. The “model-free” formalism (104, 105) is the most widespread method to study amplitude and timescale of internal bond vector motions (19). These fast protein motions can reflect the protein’s intrinsic flexibility, such as bond vibrations and side-chain rotations (19). POKY also incorporates scripts for specialized experiments. Hydrogen–deuterium exchange analysis, which monitors the rate at which amide protons swap with deuterium from the solvent on the seconds-to-hours scale, can provide a detailed map that highlights regions that are well protected and those that are exposed or involved in slow unfolding events (19). For large proteins, solvent-exposed amide-transverse relaxation-optimized spectroscopy analysis can help identify solvent-exposed amide groups, which maps surfaces of large molecular weights and their interaction interfaces (106).

Finally, POKY has visualization tools to study biomolecular interactions. The titration plot (“*ni*”) and perturbation plot (“*np*”) can directly monitor CSPs in NMR spectra as a ligand is introduced. The titration plot tool (“*ni*”) can create a binding curve plot to show how chemical shift changes as ligand concentration increases. The plot is then fitted to a binding equation to precisely calculate the dissociation constant (*K*_*d*_), which is a quantitative measure of binding strength. The perturbation plot tool (“*np*”) can show a clear visual map of a binding event by comparing the chosen observable variable, such as line width, data height, and so on, in the spectra before and after addition of ligand to a protein sample. The magnitude of the chemical shift changes is colored on the related residues when visualizing the 3D structure of the protein in PyMOL (107). In addition, CHEmical Shift Projection Analysis (108, 109) and CHEmical Shift Covariance Analysis (109, 110) are available in the POKY suite to understand the pathways of allosteric transitions in biological macromolecules based on correlated NMR chemical shift changes. With all these analyses in one unified platform, POKY can seamlessly connect atomic-level dynamics across multiple timescales.

## Metabolomics

**Figure 5:**
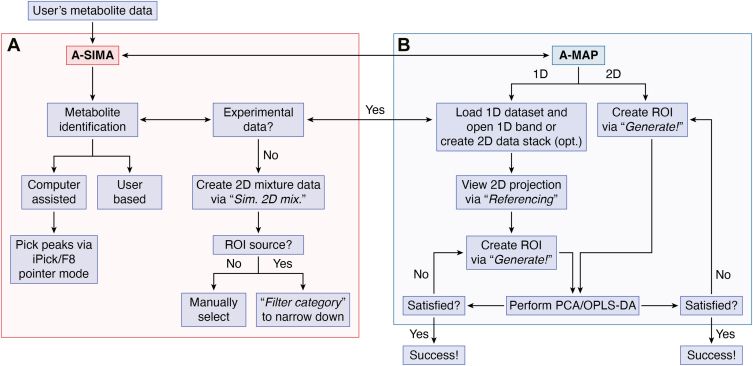
**Suggested workflow of A-SIMA and A-MAP.** Depending on the type of study, users may begin either with identification or statistical analysis. *A*, A-SIMA can be used to perform metabolite identification of 1D/2D NMR data. Identification can either be done semiautomatically or manually in A-SIMA. If user lacks experimental data, a spectrum of 2D mixture can be simulated in A-SIMA. *B*, A-MAP can be used for metabolite quantification and multivariate analysis on 1D/2D NMR data before or after identification. For input, data height, volume, or 1D array of NMR data within user-specified region of interest (ROI) will be used to perform PCA or orthogonal partial least squares-discriminant analysis. A-MAP, A Multivariate Analysis Program; A-SIMA, Advanced Software for Interactive Metabolite Analysis; PCA, principal component analysis.

Apart from proteomics studies, NMR is also applicable to the rising metabolomics field. Like protein studies, the range of available toolkits for metabolomic analysis is also quite scattered. A study on the classification of natural lignin using a variety of tools demonstrates this challenge (63). The authors used a complex, multistep workflow for their analysis, which is detailed in their methods (63). This process involved POKY for initial data processing, MATLAB for preprocessing and data conversion, MarkerView for principal component analysis, and OriginPro 2019b software for their hierarchical cluster analysis (63). This reliance on a chain of different software packages highlights a common hurdle in the field that can be difficult for nonexperts. Interestingly, the POKY suite, the software that the authors of the natural lignin study used for initial processing, has recently expanded into metabolomics with the development of two new programs, Advanced Software for Interactive Metabolite Analysis (A-SIMA) and A Multivariate Analysis Program (A-MAP) (111). Figure 5 shows an overall workflow of A-SIMA and A-MAP (111).

A-SIMA features an intuitive graphical user interface for seamless metabolite identification from 1D and 2D NMR data with a simple click of a button. A-SIMA integrates a curated library set of 2D ^1^H,^13^C-HSQC NMR metabolite spectra and 1D peak list sourced from the Biological Magnetic Resonance Bank (77), MetaLib. All compounds are organized by origin, chemical structure, and Kyoto Encyclopedia of Genes and Genomes pathways (112). A-SIMA also offers two identification modes: "Computer-Assisted" and "User-Based." In “Computer-Assisted” mode, the software groups selected peaks likely from the same compound *via* HSQCcos and queried them against MetaLib, providing likelihood values for candidates to facilitate a predict-and-confirm identification method (113). “User-Based” mode allows users to manually browse through MetaLib for direct identification. A-MAP, the second program, complements this process by providing statistical tools for metabolomics data interpretation. Users can load 1D or 2D datasets, define regions of interest, and apply preprocessing methods to extract quantitative profiles. A-MAP supports both multivariate and univariate analyses, including principal component analysis for dimension reduction and clustering, hierarchical cluster analysis, and orthogonal partial least squares-discriminant analysis for classification and validation of metabolite groups. This allows users to not only identify metabolites but also explore biological variation, detect biomarkers, and validate statistical significance. Together, A-SIMA and A-MAP offer an integrated workflow that not only brings meaningful biological interpretation to raw NMR spectra but also lowers the barriers to metabolomics research.

## Sustainability, security, and sharing

**Figure 6:**
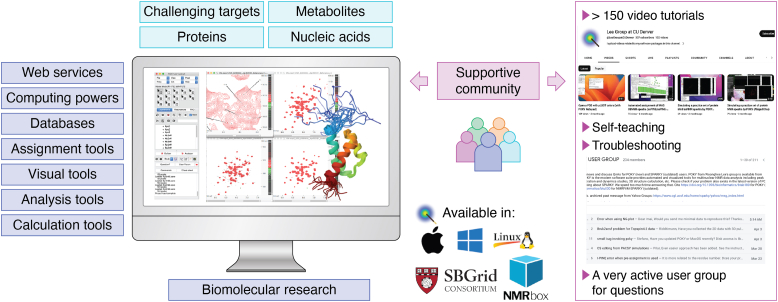
**POKY integrated platform that is available in various platforms.** There are video tutorials and active group to support accessibility.

Publicly accessible resources are critical to lower barriers to technology transfer and encourage broader adaptation. The POKY suite exemplifies this by providing availability across multiple platforms, including Apple Silicon, Apple Intel, Windows, and Linux (Fig. 6). While native installation on some specific platforms like ARM Windows/Linux and Chromebooks is limited, there are established third-party service providers, such as NMRBox (114) that can overcome this issue *via* virtual network connection technologies. SBGrid is another third-party service provider, also offering POKY as a ready-to-run software application from its software collections (115). To further support accessibility, the Lee group has created more than 150 tutorial videos hosted on their YouTube page as of November 2025. The videos cover everything from basic workflow to advanced analyses. There is also an active user group that shares troubleshooting strategies and tips (NMR POKY/SPARKY USER GROUP; https://groups.google.com/g/nmr-sparky). To support long-term sustainability, POKY provides secure and streamlined web services. It has a dedicated framework, *ServerSide App*, to simplify development, deployment, and integration of web-based tools (116). Together, this ensures that POKY remains accessible, reliable, and adaptable to help advances in computational NMR while making these technologies broadly available to scientific communities.

## Conclusion

In this review, we have highlighted how the POKY suite addresses key challenges in biomolecular NMR by providing an integrated and accessible platform for comprehensive data analysis. We have described the capabilities of NMR from spectral processing to automated resonance assignment for studying protein dynamics, biomolecular interactions, proteomics, and metabolomics. Furthermore, the process of structure calculation is streamlined using engines like PONDEROSA-C/S with database-assisted algorithms like AUDANA. With the addition of AI-based predictors like AlphaFold2/3, POKY supports a compelling workflow for hybrid structural biology, where computational models and experimental data are used cohesively. Ultimately, the POKY suite represents more than a platform for various biomolecular NMR tools. It reflects how experimental science can evolve in step with AI-driven advances. By unifying these diverse workflows, POKY enhances both accessibility and efficiency in NMR research. Its future potential reaches beyond traditional applications, such as generative AI like ChatGPT or Gemini. This generative AI could function like an intelligent research assistant to offer troubleshooting tips, suggest relevant literature, and propose new directions based on data. As more NMR-related literature is collected and used to train these AI models, these capabilities will continue to grow. In this vision, POKY not only serves as a practical tool for current research but also as an adaptable cyber infrastructure that can take on increasingly complex problems in structural biology, drug discovery, and systems-level molecular science.

## Conflict of interest

The authors declare that they have no conflicts of interest with the contents of this article.
